# A MIXED-METHODS STUDY ON THE INFLUENCE OF CHINESE CULTURE ON CAREGIVING AND IMPACTS OF CSMP

**DOI:** 10.1093/geroni/igz038.2012

**Published:** 2019-11-08

**Authors:** Jinli Wu, Mandong Liu, Geng Zhang, Iris Chi

**Affiliations:** University of Southern California, Los Angeles, California, United States

## Abstract

As a pilot study, the CSMP utilizes a mixed-methods design to tackle a lack of previous evidence and form a well-rounded perspective, using a randomized controlled trial with pre, post and 3-month follow-up assessments and focus group discussions. The CSMP not only targets cultural practices that exacerbates caregiving burden but also forms its basis upon Chinese culture and philosophies. New cultural factors together with previously-identified ones surfaced in discussions, including guanxi (Chinese relationship), renqing (favor), mianzi (self-esteem), social status, respect of authority, communication pattern, age respect, and more. The implications of cultural factors are discussed, together with corresponding areas for future improvement in CSMP and suggestions for professions working with this population. On the other side of the coin, participants who finished the CSMP achieved statistically significantly lower depression level, higher self-efficacy, and lower caregiver burden, compared to the control group.

